# Quantitative trait loci (QTL) associated with resistance of rainbow trout *Oncorhynchus mykiss* against the parasitic ciliate *Ichthyophthirius multifiliis*

**DOI:** 10.1111/jfd.13264

**Published:** 2020-09-17

**Authors:** R Jaafar, J Ødegård, H Mathiessen, A M Karami, M H Marana, L von Gersdorff Jørgensen, S Zuo, T Nielsen, P W Kania, K Buchmann

**Affiliations:** ^1^ Laboratory of Aquatic Pathobiology Department of Veterinary and Animal Sciences Faculty of Health and Medical Sciences University of Copenhagen Frederiksberg C. Denmark; ^2^ Aquagen Aas Norway; ^3^ Aquasearch ova ApS Jelling Denmark

**Keywords:** disease, quantitative trait loci, resistance, selective breeding, susceptibility

## Abstract

The parasitic ciliate *Ichthyophthirius multifiliis* has a low host specificity eliciting white spot disease (WSD) in a wide range of freshwater fishes worldwide. The parasite multiplies rapidly whereby the infection may reach problematic levels in a host population within a few days. The parasite targets both wild and cultured fish but the huge economic impact of the protozoan is associated with mortality, morbidity and treatment in aquacultural enterprises. We have investigated the potential for genetic selection of WSD‐resistant strains of rainbow trout. Applying the DNA typing system Affymetrix^®^ and characterizing the genome of the individual fish by use of 57,501 single nucleotide polymorphisms (SNP) and their location on the rainbow trout chromosomes, we have genetically characterized rainbow trout with different levels of natural resistance towards WSD. Quantitative trait loci (QTL) used for the selection of breeders with specific markers for resistance are reported. We found a significant association between resistance towards *I. multifiliis* infection and SNP markers located on the two specific rainbow trout chromosomes Omy 16 and Omy 17. Comparing the expression of immune‐related genes in fish—with and without clinical signs—we recorded no significant difference. However, trout surviving the infection showed high expression levels of genes encoding IgT, T‐cell receptor TCRβ, C3, cathelicidins 1 and 2 and SAA, suggesting these genes to be associated with protection.

## 1 INTRODUCTION

The parasitic ciliate *Ichthyophthirius multifiliis* Fouquet, 1876 has a low host specificity eliciting white spot disease (WSD) in a wide range of freshwater fishes worldwide (Matthews, 2005). The infective stage, the theront, penetrates the epithelia lining gills, skin and fins of the fish and develops into the skin feeding trophont stage which eventually ruptures the fish surface and escapes to the water as a tomont. This stage encysts and multiplies rapidly releasing numerous theronts (Li & Buchmann, 2001; Wagner, 1960) whereby the infection may reach problematic levels in the host population within a few days (Buchmann, 2019; Hines & Spira, 1974; Matthews, 2005). The parasite targets both wild and cultured fish but the huge economic impact of the protozoan is associated with mortality, morbidity, prevention and treatment in aquaculture facilities (Dickerson & Findly, 2017). The infected fish may mount a protective immune response (Bauer, 1953; Burkart, Clark, & Dickerson, 1990; Buschkiel, 1910; Dickerson & Clark, 1998; Goven, Dawe, & Gratzek, 1980; Hines & Spira, 1974; Moreira, Shoemaker, Zhang, & Xu, 2016; Sigh & Buchmann, 2001; Wang, Yu, Zhang, & Xu, 2019), but the experimental vaccines investigated so far (Burkart et al., 1990; He et al., 1997; Jørgensen et al., 2017; Ling, Sin, & Lam, 1993; Martins, Xu, Shoemaker, & Klesius, 2011; Wang, Clark, Noe, & Dickerson, 2002; Xu, Zhang, Shoemaker, & Beck, 2019) have not yet resulted in a marketable protective formulation. A number of chemotherapeutants have been successfully applied in the laboratory (Al‐Jubury et al., 2018; Picon‐Camacho, Marcos‐Lopez, & Shinn, 2012), but at farm level frequent usage of formalin, peracetic acid, sodium percarbonate and hydrogen peroxide remains the preferred control strategy (Heinecke & Buchmann, 2009; Meinelt et al., 2009; Rach, Gaikowski, & Ramsay, 2000). A genetic component associated with susceptibility and resistance to infection has been suggested in rainbowfish (Gleeson, McCalum, & Owens, 2000), leaving relevant possibilities for genetic selection of disease‐resistant strains. Single nucleotide polymorphisms (SNPs) are abundant markers evenly distributed in the animal genome representing promising tools in genetics and breeding programmes. Based on the development of a DNA typing system characterizing the genome of the individual fish, through the detection of 57,501 SNPs and their location on the rainbow trout chromosomes (Palti et al., 2015), it is possible to perform challenge tests with a certain pathogen and genetically identify fish with different levels of natural resistance. These quantitative trait loci (QTL) are directly applicable for marker‐assisted selection of breeders with elevated resistance towards a specific pathogen. With this knowledge, it is feasible to generate offspring with a higher degree of natural resistance to infection. A series of successful studies have already identified quantitative trait loci associated with natural resistance in rainbow trout against other diseases such as VHSV (Verrier et al., 2013) and *Flavobacterium psychrophilum* (Vallejo et al., 2014; Wiens et al., 2013). In Atlantic salmon, even specific genes associated with IPNV resistance have been further characterized along with the QTL (Moen et al., 2015). The present study investigates whether rainbow trout exposed to *I. multifiliis* infection exhibit different levels of resistance and whether this trait is associated with specific SNPs. We here report a significant association between resistance and SNP markers located on two specific rainbow trout chromosomes. We also present the expression of immune‐related genes in fish with and without clinical signs and in fish surviving a massive infection pressure. Despite finding QTL for resistance to *I. multifiliis* infection, we have still not identified the responsible genes. However, we discuss whether genes upregulated in surviving fish play a role in natural resistance of rainbow trout against *I. multifiliis*.

## 2 MATERIALS AND METHODS

### 2.1 Eggs

Disinfected eyed rainbow trout eggs (outbred population from 60 families, Hallesø hatchery, Aquasearch Ova, Denmark) were received (April 2019) at a certified pathogen‐free hatchery (Bornholm salmon hatchery) in Nexø, Denmark (Xueqin, Kania, & Buchmann, 2012), where eggs were hatched at 7°C within the following two weeks.

### 2.2 Larvae, fry and juveniles

Yolk sac larvae were reared to the juvenile stage in the system containing recirculated municipal water at 12°C in 700‐L tanks (total volume 1 m^3^). Fish were fed 1% biomass per day (dry pelleted feed, INICIO 917 BioMar A/S, Denmark), and after 1,440 degree‐days (August 2019)—when reaching a body weight of 4–5 g—a total of 1559 fish (mean body weight 4.6 g and mean body length 7.6 cm) were transported to the fish infection facility at the University of Copenhagen, Frederiksberg, Denmark, 1,059 being exposed to infection and 500 as non‐infected controls. Fish were kept at a 12‐hr light/ 12‐hr dark cycle in two aerated plastic tanks (INTEX^®^, Vida XL, Denmark) measuring 260 cm × 160 cm × 65 cm and each containing 800 L of municipal water at temperature 19°C which was recycled (20 L/min) by internal biofilters (AS2012, EHEIM, Germany) applying 30% water replenishment daily. Water quality was monitored and kept constant at pH 7.6, nitrite <0.01 mg/L, nitrate <50 mg/L (Tetra GmbH, Melle, Germany) and ammonia <0.5 mg/L (Hach, Loveland, USA).

### 2.3 Acclimatization

Before infection, fish were acclimatized for 14 days at the experimental conditions and head kidney swabs of five randomly selected fish were tested on 5% blood agar plates (677, SSI Diagnostica, Denmark) in order to confirm that the fish were free from bacterial infection (Dalsgaard & Madsen, 2000) and a full parasitological examination confirmed the absence of parasites (Buchmann, 2007). Fish were fed daily (INICIO 917, BioMar A/S) at a rate of 1% biomass per day. No mortality was observed during the acclimatization period.

### 2.4 Experimental exposure

A laboratory culture of *Ichthyophthirius multifiliis* (Ich) was established based on infected rainbow trout at Nørå freshwater aquaculture farm, Jutland, Denmark (August 2019). For production of infective theronts, heavily infected rainbow trout were killed in 300 mg/L MS222 (tricaine methane sulphonate, Sigma‐Aldrich, Denmark), transferred to a plastic tray with tank water where trophonts over 6 hr were allowed to escape the epidermis and transform into tomonts. These were collected and transferred to large Petri dishes containing 0.2‐µm filtered tank water (Darmstadt, Germany) (17°C). Tomonts attached to the dishes, transformed into tomocysts which released theronts after 36–42 hr. Theront density in the solution was estimated by microscopic counting of theronts in water subsamples (6 × 10 μl droplets), and the final parasite number (160,000) was for fish infection. Specifically, the 1,059 fish were infected by adding theronts (151 theronts per fish) to the tank with fish. Infection success was evaluated by counting (by use of a dissection microscope 40× magnification) the total number of developing trophonts on gills, fins and skin in a subsample of 5 fish after 6 days.

### 2.5 DNA typing

From each fish showing clinical signs, two circular tissue pieces (diameter 2.75 mm) of the tail fin were sampled immediately, by use of a punching scissor (AT7075, AgnThos AB, Sweden), and placed in a 1.5‐ml tube with lysis buffer (Vaxxinova.no) for subsequent DNA purification and genotyping. At the end of the experiment, surviving fish were similarly sampled. DNA typing was conducted according to Palti et al. (2015). In brief, restricted DNA from each fish was applied to the 57,501 SNPs Axiom^®^ Trout Genotyping Array in 96‐well format, with specified probes for specific SNPs evenly distributed in the rainbow genome (29 chromosomes). Genotyping was performed using Affymetrix’ proprietary Axiom platform, following the Axiom^®^ 2.0 Assay Automated Workflow (http://media.affymetrix.com/support/downloads/manuals/axiom_2_assay_auto_workflow_user_guide.pdf).

### 2.6 Sampling for gene expression

Samples of gills, liver and spleen (15 fish/group) were taken from the unchallenged fish (control fish) at different time points (day 0, 17 and 21 days post‐challenge, dpc). Correspondingly, at 17 dpc, we took samples from challenged fish with no clinical signs (NCS) and with clinical signs (CS) and finally at 21 dpc from fish surviving the challenge. Tissues were preserved in RNAlater (R0901, Sigma‐Aldrich, Denmark) and incubated (24 hr) at 4°C and then stored at −20°C until use.

### 2.7 RNA isolation, cDNA synthesis and quantitative reverse transcription PCR (qPCR)

RNA purification and cDNA synthesis were mainly performed according to Karami et al. (2018) with some modifications. Tissues (gills, liver and spleen) were homogenized (2 min, 20 Hz; Tissue lyser II, Qiagen, Denmark) using a homogenization buffer with 2‐mercaptoethanol (Sigma‐Aldrich), after which RNA was recovered by the GenEluteTM mammalian RNA kit (RTN350, Sigma‐Aldrich, Denmark). For liver samples, the Proteinase K (cat.no.P4850) protocol of the kit was used before performing the above‐described RNA extraction. DNase I (AMPD1, Sigma‐Aldrich, Denmark) treatment removed genomic DNA, and the concentration of RNA in isolated preparations was determined applying a NanoDrop 2000 spectrophotometer (Saveen & Werner, Denmark). Quality of RNA and the DNase treatment was evaluated by electrophoresis (ethidium bromide‐stained agarose) (Invitrogen). RNA was kept at −80°C until cDNA synthesis in T100 Thermocycler (Bio‐Rad, Denmark) using a 20 μl reaction volume with 1,000 ng of RNA, oligo d(T)16 primer and TaqMan^®^ reverse transcription reagents (N8080234, Thermo Fischer Scientific, Denmark) (25°C for 10 min, 37°C for 60 min, 95°C for 5 min). Finally, cDNA (10 × diluted to 200 μl with RNase‐free H2O (10977049, Thermo Fischer Scientific, Denmark) was stored at −20°C until further use. Gene expression analyses were slightly modified from Karami et al. (2018). Quantitative PCR assays were performed using an AriaMx Real‐Time PCR machine (G8830A‐04R‐010, AH diagnostics). The cycling conditions were one cycle of predenaturation at 95°C for 15 min. This was followed by 40 cycles of denaturation at 94°C for 10 s with a combined annealing/elongation process at 60°C for 45 s with endpoint measurement. Primers and TaqMan probes (synthesized at TAG Copenhagen AS, Denmark) for immune‐relevant rainbow trout genes are shown in Table S1. Reaction volumes were 12.5 μl (2.5 μl cDNA, 6.25 μl Brilliant III Ultra‐Fast QPCR Master Mix (600881, AH Diagnostics AS, Denmark), 1.0 μl primer‐probe mixture (forward primer, 10 μM and reverse primer, 10 μM), TaqMan probe (5 μM) and 2.75 μl RNase‐free water. Reverse transcriptase minus and negative controls were used for every plate setup. Using NormFinder (Andersen, Jensen, & Ørntoft, 2004) and all combinations of three genes encoding elongation factor (ELF) 1‐α, β‐actin and ARP genes, the average of the three genes was found suitable as endogenous control (reference genes). Stability values were 0.002, 0.005 and 0.002 for liver, spleen and gills, respectively.

Investigated genes encoding immune‐relevant molecules (Table S1) included interleukins (IL‐1β, IL‐2A, IL‐4/13A, IL‐6A, IL‐8 (isoforms A to E), IL‐10A, IL‐12‐α chain, IL‐17A/F2A, IL‐17C1, IL‐17C2, IL‐22), type II interferon (IFN‐γ1 and IFN‐γ2), transforming growth factor‐β (TGF‐β1a), tumour necrosis factor‐α (TNF‐α), serum amyloid protein A (SAA), complement factor 3 isoform 4 (C3‐4), immunoglobulins (IgM, IgT, IgDm, IgDs), cathelicidins (cathelicidin 1, cathelicidin 2), T‐cell receptor‐β (TCR‐β) and lysozyme.

The parasite infection level from 17 dpc was estimated by quantifying the expression of the gene encoding the *Ichthyophthirius multifiliis IAG52A* surface antigen. Primers and probe were designed in this study using Primer3Plus v2.4.2 (Untergasser et al., 2007). The specificity of the assay was confirmed by performing PCR using a plasmid containing the *IAG52B* gene. The efficiency (103%) was determined by serial dilutions of the parasite template.

### 2.8 Data analysis

Cumulative mortality rates were analysed by the Kaplan–Meier survival analysis (GraphPad Prism version 4, Bethesda, ML, USA).

Gene expression was analysed by the simplified 2^–ΔΔCq^ method (Livak & Schmittgen, 2001) with all qPCR assays having efficiencies within the range 100% ±5%.

At each time point, all challenged fish groups were compared to non‐exposed controls using Student's *t* test. Only regulations with a fold change >2 and *p* < .05 were considered significant. Less than three Cq values were obtained in the time point controls for 4 genes. In those cases, a qualitative assessment (presence/absence of Cq value) was used and analysed with the nonparametric Mann–Whitney U test using a probability level of 5%. Pearson's correlation test was used for analysing the correlation between the parasite load and the expression of specific immune genes in gills of trout.

The morbidity/mortality of challenged fish during the test was high (98%), and the phenotype used in genetic analyses was thus time (hours) to morbidity/death, using the GCTA software (Yang, Lee, Goddard, Visscher, & Peter, 2011). The survivors were given an artificial survival time of 24 hr past the last recorded mortality. A genomic animal model was used for estimation of genetic variance and heritability (GREML). The model was as follows:

y=Xμ+g+e

where **y** is a vector of phenotypes (time to morbidity/death), μ is a vector of tank effects (with associated incidence matrix **X**), $g∼N(0,G\sigma_{g}^{2})$ is a vector of random additive polygenic effects, and $e∼N(0,I\sigma_{e}^{2})$ is a vector or random residuals. The matrix G is the genomic relationship matrix (Van Raden, 2008):

$$G=\frac{1}{\sum 2p_{i}\left(1‐p_{i}\right)}{\mathrm{MM}}^{\prime }$$

where M is a centred genotype matrix (one column per locus, one line per individual) and $p_{i}$ is the allele frequency for locus *i*. Subsequently, a similar model was also used for the genomewide association study (GWAS), accounting for single SNP effects as well as polygenic effects, using the leave‐one‐chromosome‐out option (LMM‐LOCO) for the latter. This implies that SNPs from the chromosome currently tested are excluded in computation of the GRM. In general, the SNPs used in the analysis were restricted to those of high genotype quality (“PolyHighRes”) and MAF > 0.01, resulting in 33,963 SNPs being used in the analysis. A total of 1,059 fish of high genotype quality was included in the analysis.

### 2.9 Ethics and legislation

Infection procedures were performed under the licence 2019‐15‐0201‐01614 obtained from the Experimental Animal Inspectorate, Committee for Experimental Animals, Ministry of Environment and Food, Denmark, complying with EU Directive 2010/63/EU. Fish were observed every 2nd hour according to the ethical guidelines of the University of Copenhagen. Death was not used as humane endpoint but fish showing clinical signs (loss of equilibrium, irregular swimming, discoloration) were taken out and killed in an overdose MS222 (tricaine methane sulphonate, Sigma‐Aldrich, Denmark; 300 mg/L) and recorded as mortality.

## 3 RESULTS

### 3.1 Infection success

The number of trophonts in the skin, fins and gills was counted to 5.5 trophonts per fish (infection success 3.6%) on day 6 post‐challenge. From 13 dpc, numerous white spots were visible on the fish, and from 17 dpc, the level of parasitization was too high to count the exact number of trophonts. Instead, the relative intensity was estimated by molecular means (number of transcripts of the parasite i‐antigen *IAG52A*).

### 3.2 Morbidity/mortality

**Figure 1:**
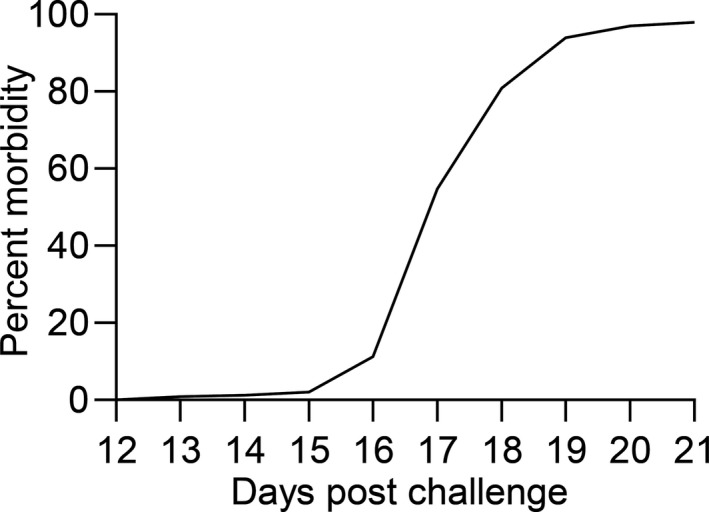
Morbidity/mortality curve following exposure of 1,059 rainbow trout fry to *Ichthyophthirius multifiliis* (Ich)

The first episode of morbidity/mortality was recorded at 13 dpc and the number increased exponentially until morbidity/mortality peaked around 17 dpc and levelled off at day 19 (Figure 1). Overall morbidity/mortality was 98% with a mean survival time of 120.5 hr (Table 1). Non‐infected controls showed 100% survival.

**Table 1:** Survival data

Trait	Mean	*SD*
Survival at end of exposure period	2%	0.14
Survival time (hr)	120.5	29.9

### 3.3 Genetic analysis

**Figure 2:**
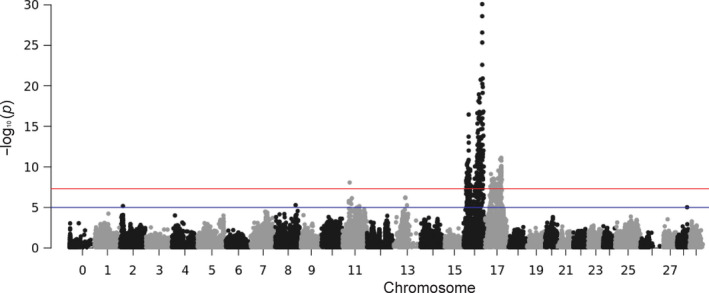
Manhattan plot from a LMM‐LOCO‐GWAS of time to disease/death in rainbow trout after exposure to *Ichthyophthirius multifiliis* infection [Colour figure can be viewed at wileyonlinelibrary.com]

The results from the REML show that the heritability was extremely high (0.58 ± 0.04), but still trustworthy (only high‐quality SNPs were used) (Table 2). The resulting Manhattan plot from the LOCO‐GWAS is presented in Figure 2. The results indicate a highly significant QTL on chromosome 16 and a significant QTL on chromosome 17. A possible QTL was also suggested on chromosome 11. As seen from Table 3, the most significant SNP apparently explains a substantial part of genetic variance. Still, as nearly all fish eventually reached morbidity, the QTL mainly affect time to morbidity/mortality (16 hr extra per resistance allele) rather than whether or not the fish dies, at least under experimental conditions. The allele frequency of the favourable allele seems to be moderate (~30%). As a consequence, only ~10% of the fish in the current population are expected to be homozygous for the favourable allele.

**Table 2:** Estimated variance components from REML analysis of a genomic animal model

Factor	Estimate	SE
Additive genetic variance	502.178452	65.501182
Residual variance	361.656894	24.987859
Phenotypic variance	863.835347	56.593752
Heritability	0.581336	0.042999

**Table 3:** The top SNPs at chromosome 16 and 17. The two last columns give the estimated fraction of genetic and phenotypic variance explained by each SNP

Chromosome	SNP	Position	Type	Frequency	Effect	*SE*	*p*	Fraction genotypic variance	Fraction phenotypic variance (%)
16	AX−89960822	60496258	C/A	0.66	−16.1	1.4	8.28E−31	23%	13%
16	AX−89934892	60498371	C/T	0.74	−17.1	1.5	2.53E−29	22%	13%
16	AX−89936665	60515930	C/T	0.77	−17.3	1.6	2.68E−27	21%	12%
16	AX−89976110	60609017	G/T	0.26	16.4	1.5	4.56E−26	21%	12%
16	AX−89916752	60556546	C/T	0.63	14.0	1.4	2.57E−23	18%	11%
17	AX−89923139	52080013	G/A	0.46	8.8	1.3	7.59E−12	8%	4%
17	AX−89957772	48828265	C/A	0.43	9.0	1.3	1.17E−11	8%	5%
17	AX−89947214	52422379	G/A	0.47	9.0	1.3	1.69E−11	8%	5%

### 3.4 Parasite gene expression (parasite load)

**Figure 3:**
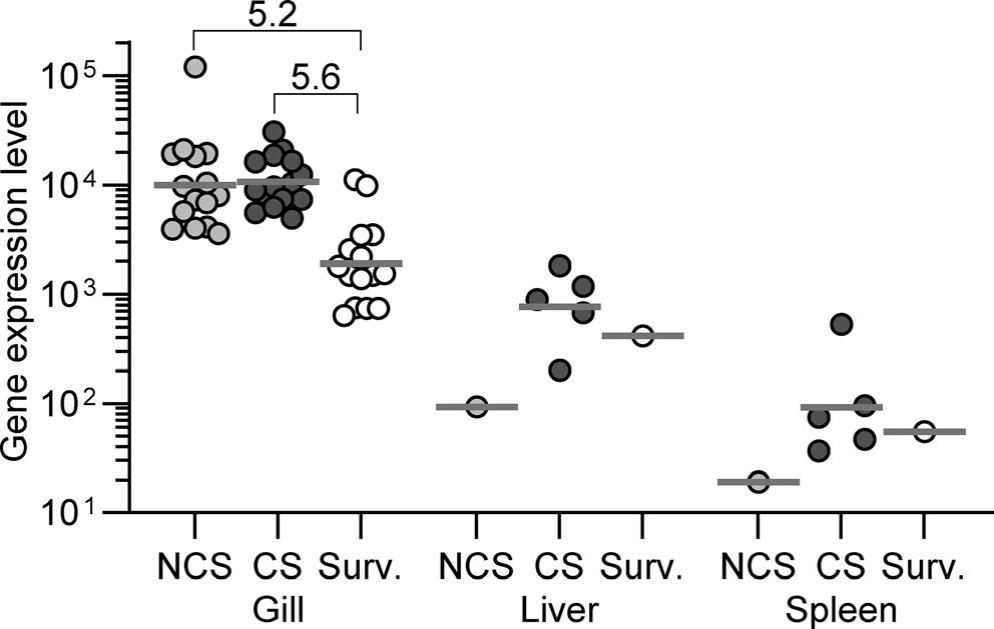
Parasite infection levels at 17 and 21 dpc expressed as the relative transcript levels in different organs of the gene encoding the *I. multifiliis* i‐antigen *IAG52A* in rainbow. Transcript levels in highly susceptible fish showing clinical signs (CS) at 17 dpc, as well as less susceptible fish showing no clinical signs (NCS) at the same time point and surviving fish at 21 dpc

The expression of the *IAG52A* gene in the gills was significantly (fivefold) higher in diseased fish (NCS and CS) compared with surviving fish (Figure 3). The expression of the parasite gene was detected in few spleen and liver samples of fish mainly showing clinical signs.

### 3.5 Rainbow trout immune gene expression

**Figure 4:**
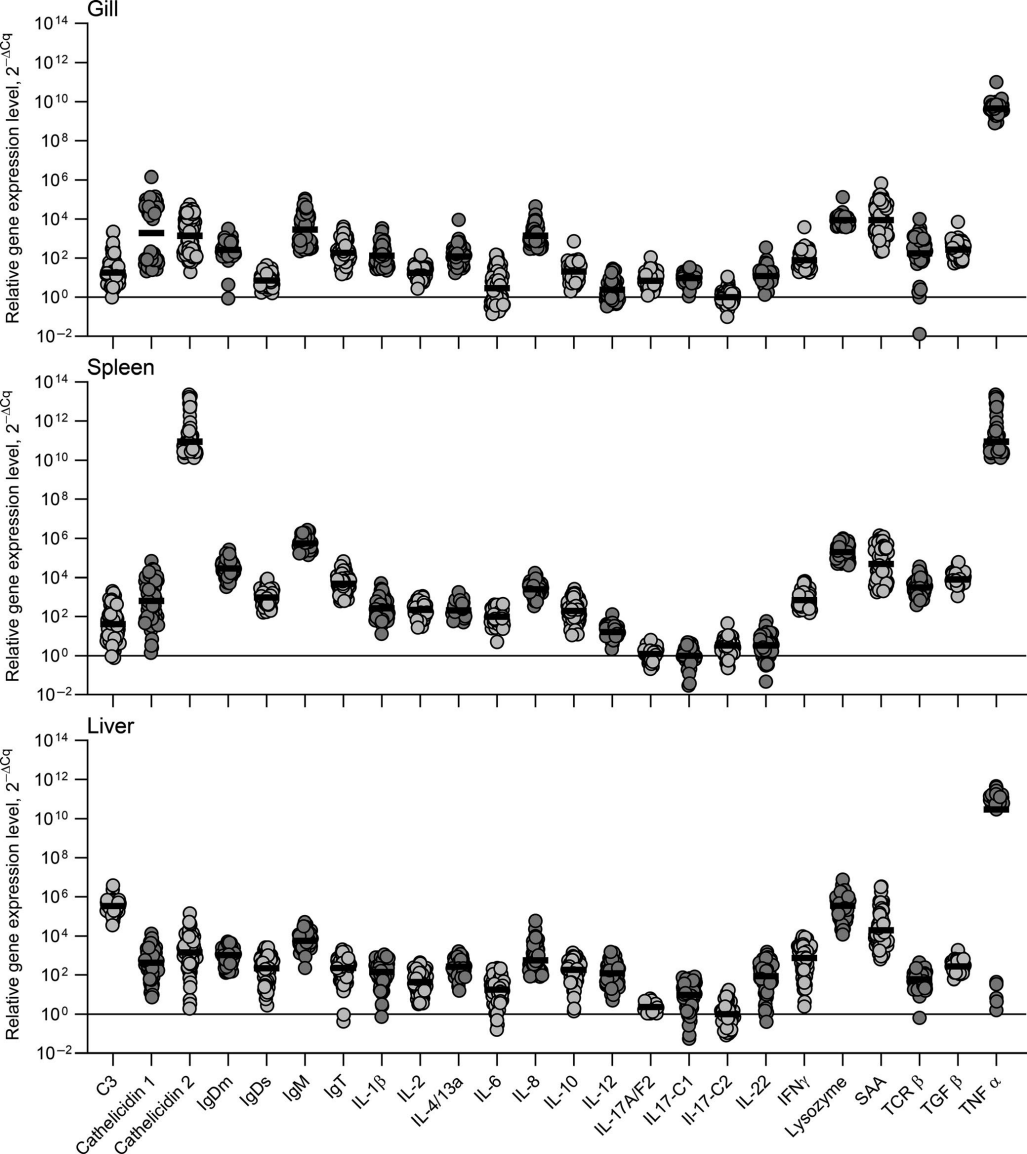
Relative expression levels of immune‐relevant genes (all fish included)

**Figure 5:**
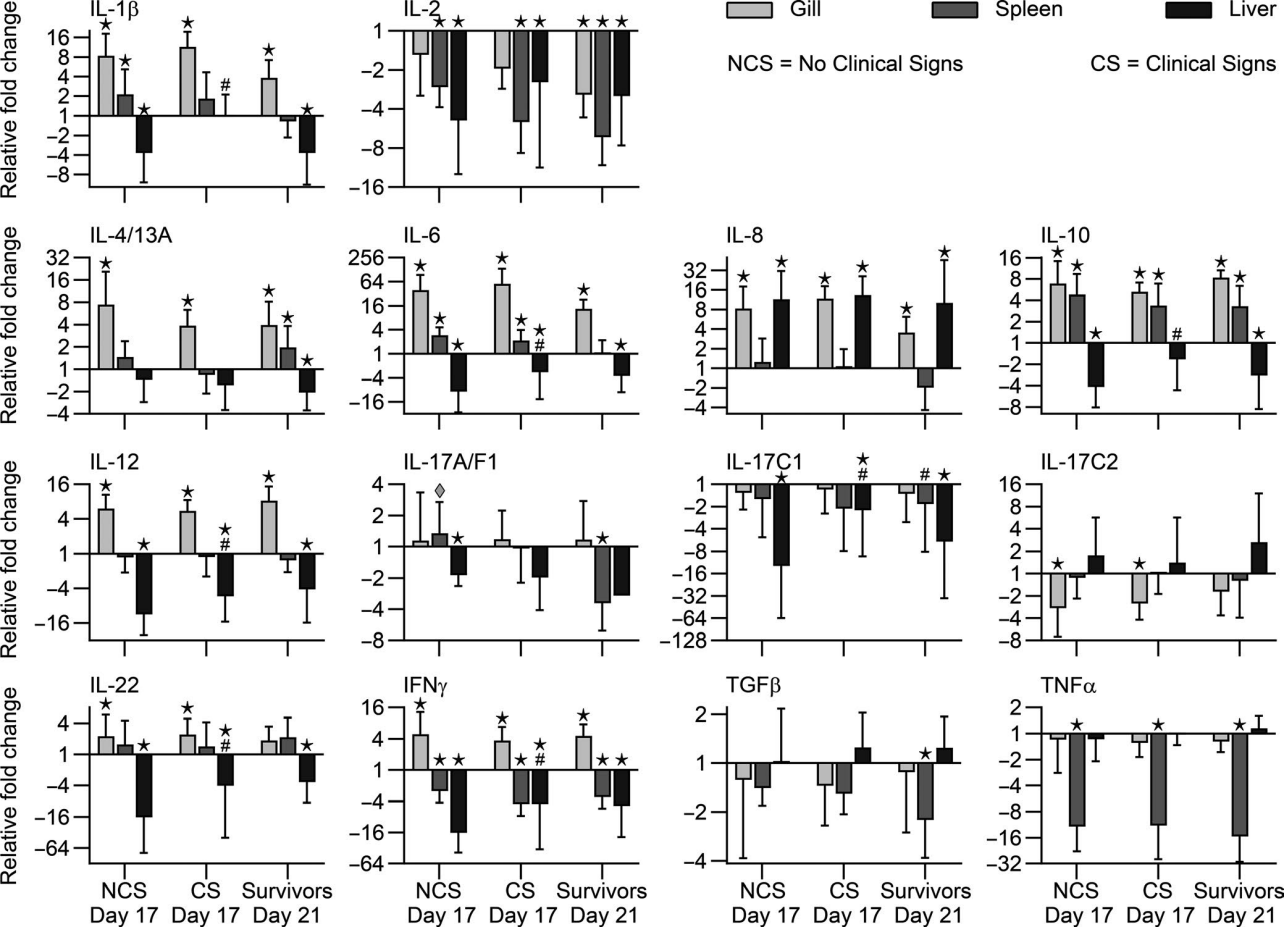
Expression of immune‐relevant cytokine genes in rainbow trout organs (gills, spleen, liver) following exposure to *I. multifiliis* at different time points. Relative fold changes in relation to non‐exposed time point controls for 1) highly susceptible fish showing clinical signs (CS) at 17 dpc. compared to 2) less susceptible fish showing no clinical signs (NCS) at the same time point and 3) surviving fish at 21 dpc

**Figure 6:**
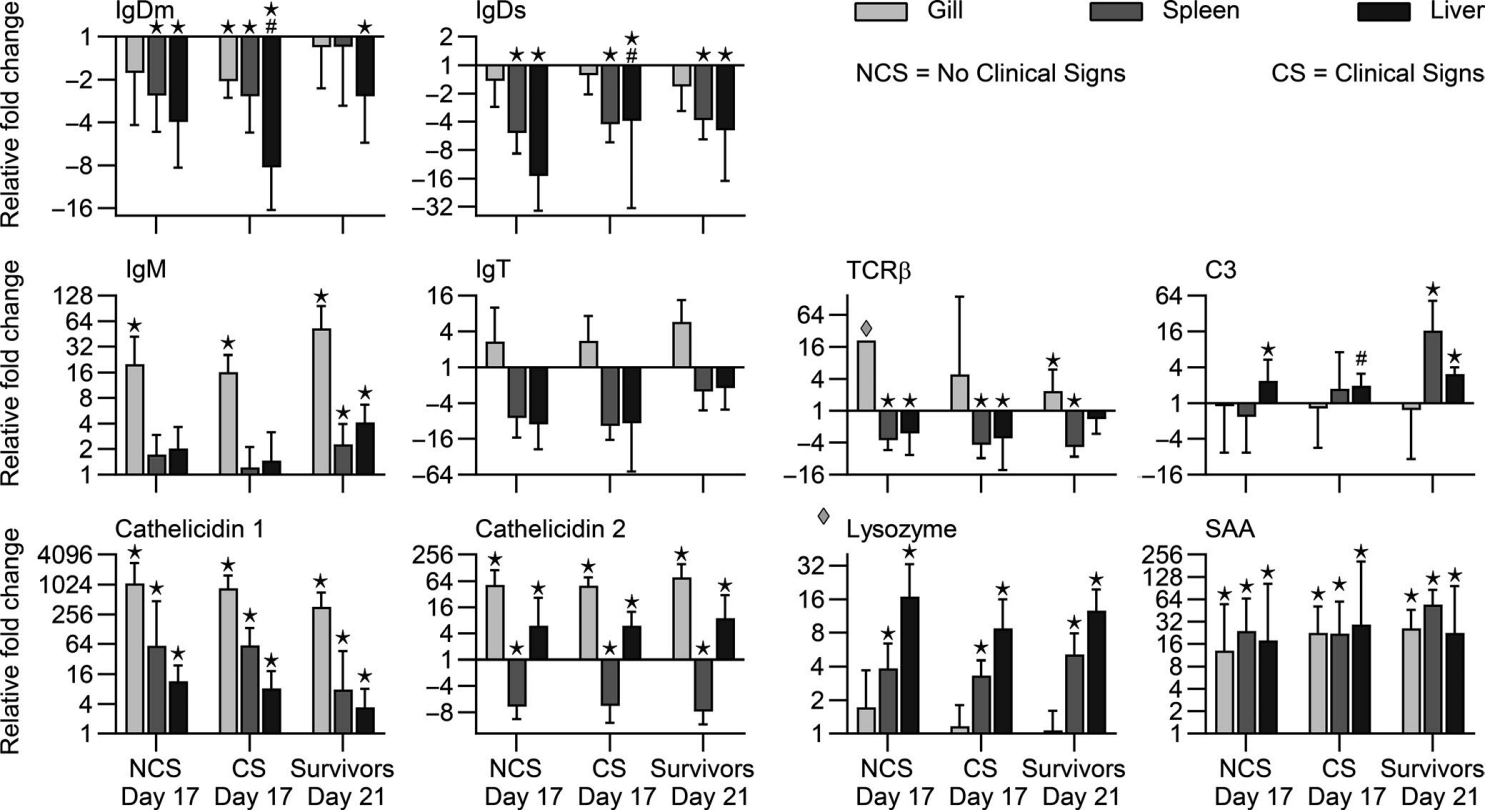
Expression of immune‐relevant effector molecules encoding genes in rainbow trout (gills, spleen, liver) infected with *I. multifiliis*. Relative fold changes in relation to non‐exposed time point controls for 1) highly susceptible fish showing clinical signs (CS) at 17 dpc. compared to 2) less susceptible fish showing no clinical signs (NCS) at the same time point and 3) surviving fish at 21 dpc

The overall expression levels of genes in all trout (exposed and non‐exposed) are displayed in Figure 4 with details in Table S2. Genes encoding immune‐relevant molecules were expressed to different degrees, but they were generally upregulated in the gills of fish exposed to parasites when compared to non‐exposed control fish. The expression levels of genes encoding cytokines (IL‐1β, IL‐4/13A, IL‐12, IL‐22, IL‐2, IL‐6, IL‐17A/F2, IFN‐γ, IL‐8, IL‐17C1, TGF‐β, IL‐10, IL‐17C2 and TNF‐α) in gills, spleen and liver of the infected fish groups (NCS, CS and survivors) are shown in Figure 5. The gills and spleen of NCS, CS and surviving fish showed significantly higher expression of cytokine genes. In contrary, all cytokine genes, except those encoding IL‐8, TGF‐β, IL‐17C2 and TNF‐α, were significantly downregulated in liver of all challenged fish (Figure 5). The expression of genes encoding effector molecules including IgDs, IgDm, IgM, IgT, cathelidin‐1, cathelidin‐2, TCRβ, lysozyme, C3 and SAA resulted different in the three organs (Figure 6). IgDm and IgDs genes were significantly downregulated in all organs of NCS, CS and surviving fish. The IgT gene was slightly upregulated in the gills of all fish groups, whereas it was downregulated in spleen and liver (Figure 6). The gene expression of IgM was elevated in the gills of all fish and in the internal organs of surviving fish. The expression of cathelidin‐1 and SAA genes was significantly upregulated in the gills, spleen and liver of all the challenged groups, whereas cathelicidin 2 gene expression was upregulated in gills and liver. The gene encoding lysozyme was significantly upregulated in spleen and liver—but not in gills—of all fish groups. The C3 gene was particularly upregulated in spleen and liver in surviving fish. The TCR‐β gene expression was significantly upregulated in the gills of all infected fish but resulted downregulated in the internal organs (Figure 6).

### 3.6 Correlation between parasite load and immune gene expression

**Figure 7:**
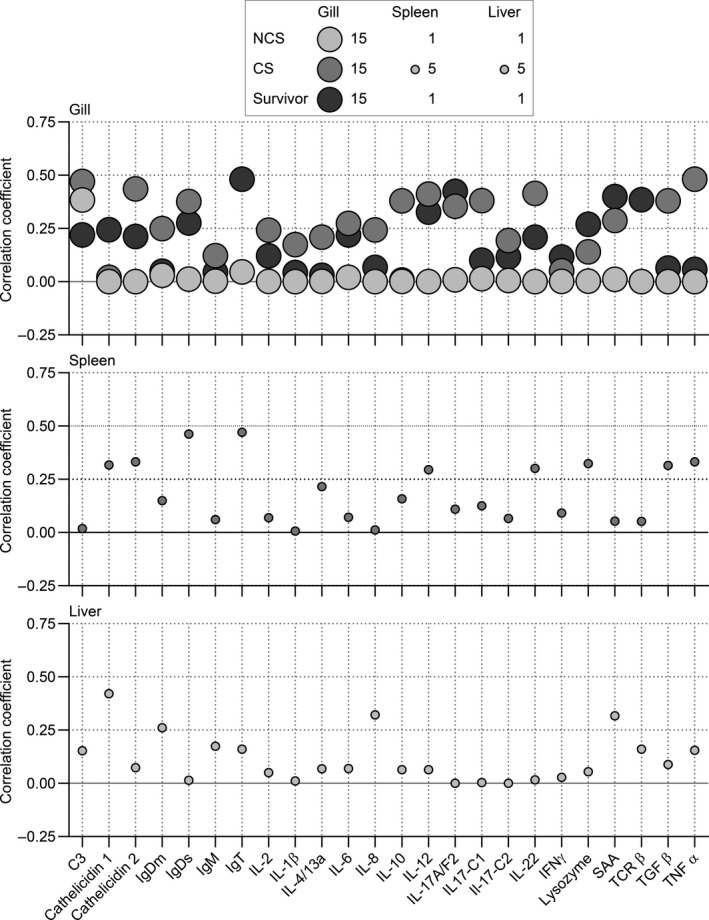
Correlation coefficients between parasite load (expressed as the relative transcript levels of the gene encoding the *I. multifiliis* i‐antigen *IAG52A)* and the expression of specific immune genes in trout. For the three fish groups: 1) highly susceptible fish showing clinical signs (CS) at 17 dpc; 2) less susceptible fish showing no clinical signs (NCS) at the same time point and 3) surviving fish at 21 dpc

Parasite transcripts were found in the gills of all the exposed fish, whereas only a few fish showed parasite transcripts in the internal organs (spleen and liver) whereby the correlation factors are non‐significant. Positive correlations between parasite loads and gill expression levels of all immune genes were found in CS and surviving fish (Figure 7). In NCS, only the gene encoding complement factor C3 was regulated in accordance with the infection level. The correlation in surviving fish was particularly high for the genes encoding IgT, TCRβ, SAA, C3 and cathelicidins 1 and 2.

## 4 DISCUSSION

The protozoan *Ichthyophthirius multifiliis* is generally considered a highly pathogenic parasite (Matthews, 2005), and the present investigation confirmed that the infection—if left untreated or unmanaged—leads to high morbidity—in this case 98% of the infected fish population—within a few weeks (21 days). From a limited experimental exposure using 151 theronts per fish, we observed that 3.6% established as trophonts in the fish surface within one week but then the parasite demonstrated a strong potential of multiplication. Already from day 13, we recorded the first severe disease signs in fish—apart from the visible white spots on the fish surface. When estimating the infection by molecular tools (expression of the parasite i‐antigen in gills, spleen and liver), the infection was severe in the infected fish (with and without signs) and lowest in surviving fish. The presence of parasite transcripts in only a few internal organs in fish showing clinical signs demonstrated that the parasite targets epidermal sites only. If the presence of parasite transcripts in internal organs is due to a systemic infection associated with the high infection pressure or should be interpreted as antigen‐presenting cells in gills (Kato et al., 2018), transporting parasite elements to spleen should be further investigated. Some fish were more susceptible than others to *I. multifiliis* infection. Some fish were moribund whereas others in the same fish tank appeared unaffected. Genetic factors could explain this difference which would support previous observations (Gleeson et al., 2000), and therefore, we investigated this possibility. If markers associated with natural resistance can be identified, they can be applied in breeding programmes (Gjedrem & Baranski, 2009). Single nucleotide polymorphisms (SNP) linked to genes encoding specific traits (QTL, quantitative trait loci) may find further application in marker‐assisted selection (MAS) (Palti et al., 2015). By the advent of the SNP genotyping array, it has been possible to analyse a high number of rainbow trout with different traits. The array contains detection abilities for 57,501 SNPs which are well represented throughout the rainbow genome on 29 chromosomes (Palti et al., 2015). When fish were sampled at the first sign of disease throughout the observation period and subsequently subjected to SNP analysis (Affymetrix^®^), the most significant SNP explains a substantial part of genetic variance. The QTL found on chromosomes 16 and 17 were highly significant. Still, as nearly all fish die, the QTL mainly affect time of mortality (16 hr extra per resistance allele for the most significant SNP) rather than whether or not the fish dies, at least under experimental conditions. Under natural infections, possibly with lower parasite load per fish, the QTL may be more likely to prevent mortality rather than merely delaying it. The frequency of the favourable allele seems to be moderate (~30%). Consequently, the potential for genetic gain is substantial. Currently, only ~10% of the fish are expected to be homozygous for the favourable allele and the present study showing QTL linked with chromosomes 16 and 17 in rainbow trout makes it possible to implement breeding of trout with improved natural resistance. Our gene expression analyses on fish with and without clinical signs showed a general upregulation of immune‐related genes, suggesting that both susceptible and less susceptible fish activate central immune mechanisms in response to infection. However, it is of special interest to analyse the genes expressed in trout surviving the constant exposure to numerous invading theronts over 21 days. The marked expression of genes encoding IgM, IgT, T‐cell receptor β, complement factor C3, lysozyme, cathelicidins 1 and 2 and SAA is noteworthy and suggests that these genes may play a role in rainbow trout immune response against *I. multifiliis* infection. The involvement of protective antibodies in fish against this ciliate has previously been established (Dickerson & Clark, 1998; Hines & Spira, 1973, 1974; Olsen et al., 2011; Xu et al., 2013). Not only IgM but in particular IgT (Hansen, Landis, & Philips, 2005; Zhang, Zhang, Chen, Sunyer, & Zhang, 2017) associated with fish mucosal immunity may play a special role (Jørgensen, Heinecke, Skjødt, Rasmussen, & Buchmann, 2011; Olsen et al., 2011; Xu, Klesius, & Shoemaker, 2008; Xu et al., 2013; Yu et al., 2018). Fish with WSD show a marked leucocyte infiltration of affected tissues (Cross & Matthews, 1993; Hines & Spira, 1973; Jørgensen, Korbut, Jeberg, Kania, & Buchmann, 2018; Ventura & Paperna, 1985). Granulocytes including neutrophils are active players but the elevated specific T‐cell reactivity in gills infected by *I. multifiliis* as demonstrated by Olsen et al. (2011) is noteworthy. Gills contain dense aggregates of T cells in interbranchial tissue (Koppang et al., 2010), and these cells react to the infection, in agreement with the elevated TCR expression in the present study.

Resistance to infection is probably based on the development of a non‐attractive and hostile environment for the invading parasite (the theront) in the fish surface. Apart from antibodies and T‐cell reactivity, complement factors, such as C3, may directly bind to the invading theront (Gonzalez, Buchmann, & Nielsen, 2007a). In addition, the pentraxin SAA possessing several binding sites for pathogens has previously been associated with a response in fish to this parasite (Gonzalez, Buchmann, & Nielsen, 2007b). Moreover, serum lysozyme was previously shown to be elevated in infected fish suggesting its role in the systemic immunity (Alishahi & Buchmann, 2006). The expression of cathelicidin genes, as observed in surviving fish in response to *I. multifiliis*, may contribute to the development of a hostile microenvironment for the invading parasites. Production of AMPs is generally observed in rainbow trout exposed to various pathogens (Furlan et al., 2018) including protozoan skin parasites (Chettri et al., 2014). Correspondingly, chemokines may have a direct lethal effect on *I. multifiliis* theronts (Munoz‐Atienza et al., 2019).

The upregulation of this range of immune genes in trout surviving an extreme and increasing infection pressure over 21 days suggests that these genes are involved in the natural resistance to parasite infection. However, recent transcriptomic and proteomic analyses of *I. multifiliis*‐infected rainbow trout show that hundreds of other genes are upregulated or transcribed as well (Saleh et al., 2019; Syahputra et al., 2019). In addition, a number of genes resulted downregulated in the present study, suggesting that the factors associated with the natural resistance to *I. multifiliis* infection may be part of an intricate and regulated system. In order to elucidate this question in further depth, future studies should map the genes located on the rainbow trout chromosomes Omy16 and Omy17 and investigate how these genes are related to the responses shown in the present work.

## CONFLICT OF INTERESTS

Aquagen is a commercial company conducting research related to genetic resources and breeding. Aquasearch ova ApS is a Danish trout breeding company.

## Supporting information

Table S1

Table S2

## Data Availability

All data from this study are available following open access publication of the article.
