# Supplementary material for: Quantitative trait loci (QTL) associated with resistance of rainbow trout Oncorhynchus mykiss against the parasitic ciliate Ichthyophthirius multifiliis
Source: J Fish Dis. 2020 Sep 17;43(12):1591–602. doi: 10.1111/jfd.13264 (PMC7692903; doi:10.1111/jfd.13264)
Supplement: Supplementary file 1 — Table S1 [file JFD-43-1591-s001.docx]

**Supplementary Table S1**

Primers and probes used for qPCR assays. All nucleotides are shown from 5’ end (labeled with FAM) to 3’ end (labeled with BHQ1). All the qPCR assays were optimized for an annealing temperature of 60°C and efficiencies of 100% ± 5%. ^R^ : reference genes (house keeping genes). ^MS^ : the qPCR assay targets both membrane bound and secreted forms of the gene product. ^1^ : The α chain of IL-12 is common to the two isoforms of IL-12.

| **Gene**  **GenBank acc.no.** | **Length**  **bp** | **Primers & Probes**  **5’end to 3’end** | **References** |
| --- | --- | --- | --- |
| *^R^ ARP*  *AY505012* | 106 | Fwd: GAAAATCATCCAATTGCTGGATG  Rev: CTTCCCACGCAAGGACAGA  Probe: CTATCCCAAATGTTTCATTGTCGGCGC | (Purcell, Kurath, Garver, Herwig and Winton, 2004) |
| *^R^ β-actin*  *AB196465* | 241 | Fwd: ACATCAAGGAGAAGCTGTGCTAC  Rev: TACGGATGTCCACGTCACAC  Probe: CCTCTCTGGAGAAGAGCTACGAGCTG | (Marana, Chettri, SaltenBach-Olesen, Kania, Dalsgaard and Buchmann, 2020) |
| *^R^ ELF-1α*  [*AF498320*](http://www.ncbi.nlm.nih.gov/entrez/viewer.fcgi?db=nucleotide&val=20269865) | 63 | Fwd: ACCCTCCTCTTGGTCGTTTC  Rev: TGATGACACCAACAGCAACA  Probe: GCTGTGCGTGACATGAGGCA | (Ingerslev, Pettersen, Jakobsen, Petersen and Wergeland, 2006) |
| *C3.3 & C3.4*  *AF271080 / U61753* | 85 | Fwd: ATTGGCCTGTCCAAAACACA  Rev: AGCTTCAGATCAAGGAAGAAGTTC  Probe: TGGAATCTGTGTGTCTGAACCCC | (Raida and Buchmann, 2009) |
| *Cathelicidin 1A*  *AY382478* | 189 | Fwd: TCTCTCGTCCTGGGGTT  Rev: GTTGTAGCGTGCTGATCTATG  Probe: TAATTGGTCGTCCTGGGGGTGG | (Marana *et al.,* 2020) |
| *Cathelicidin 2A*  *AY360356* | 135 | Fwd: AAAGATTCCAAGGGGGGT  Rev: CAAAGGGTGTGTTGTGCTGT  Probe: GCTCTCGTCCTGGGTTTGGCTCC | (Xueqin, Kania and Buchmann, 2012) |
| *IFN γ1 and IFN γ2*  *FJ184374 / FJ184375* | 68 | Fwd: AAGGGCTGTGATGTGTTTCTG  Rev: TGTACTGAGCGGCATTACTCC  Probe: TTGATGGGCTGGATGACTTTAGGA | (Raida and Buchmann, 2007) |
| *IgDm*  *AY870262* | 304 | Fwd: CAGGAGGAAAGTTCGGCATCA  Rev: CCTCAAGGAGCTCTGGTTTGGA  Probe: CCACACCACACAGACTCTGGCCCTGAA | (Skov, Chettri, Jaafar, Kania, Dalsgaard and Buchmann, 2018) |
| *IgDs*  *JQ003979* | 304 | Fwd: TGGCACGCCAGGATTTGAC  Rev: TCAGAATTGAGTGAACGGACAGACA  Probe: CCACACCACACAGACTCTGGCCCTGAA | (Skov *et al.,* 2018) |
| *^MS^ IgM*  *S63348 / AH014877* | 72 | Fwd: ACCCTCCTCTTGGTCGTTTC  Rev: TGATGACACCAACAGCAACA  Probe: TGATGACACCAACAGCAACA | (Raida and Buchmann, 2007) |
| *^MS^ IgT*  *AY870265 / AY870263* | 73 | Fwd: AGCACCAGGGTGAAACCA  Rev: GCGGTGGGTTCAGAGTCA  Probe: AGCAAGACGACCTCCAAAACAGAAC | (Raida and Buchmann, 2007) |
| *IL-1β*  *AJ223954* | 91 | Fwd: ACATTGCCAACCTCATCATCG  Rev: TTGAGCAGGTCCTTGTCCTTG  Probe: CATGGAGAGGTTAAAGGGTGGC | (Raida and Buchmann, 2007) |
| *IL-2a*  *FJ571513* | 110 | Fwd: ATGCAACACCACATCAGCAT  Rev: TGCCACGGCCCTACAAAAGA  Probe: TGCCACGGCCCTACAAAAGA  RE  TGCCACGGCCCTACAAAAGA | (Marana *et al.,* 2020) |
| *IL-4/13a*  *AB574337* | 138 | Fwd: ATCCTTCTCCTCTCTGTTGC  Rev: GAGTGTGTGTGTATTGTCCTG  Probe: CGCACCGGCAGCATAGAAGT | (Chettri, Kuhn, Jaafar, Kania, Moller and Buchmann, 2014) |
| *IL-6a*  *DQ866150* | 91 | Fwd: ACTCCCCTCTGTCACACACC  Rev: GGCAGACAGGTCCTCCACTA  Probe: CCACTGTGCTGATAGGGCTGG | (Raida and Buchmann, 2008) |
| *IL-8 isoforms a, b , c, d & e*  *AY160982 to AY160986* | 69 | Fwd: AGAATGTCAGCCAGCCTTGT  Rev: TCTCAGACTCATCCCCTCAGT  Probe: TTGTGCTCCTGGCCCTCCTGA | (Raida and Buchmann, 2008) |
| *IL-10a*  [*AB118099*](http://www.ncbi.nlm.nih.gov/entrez/viewer.fcgi?db=nucleotide&val=47678892) | 70 | Fwd: CGACTTTAAATCTCCCATCGAC  Rev: GCATTGGACGATCTCTTTCTTC  Probe: CATCGGAAACATCTTCCACGAGCT | (Raida and Buchmann, 2007) |
| *^1^ IL-12 α chain*  *HE798148* | 84 | Fwd: CAACGGAACACCACATTCAG  Rev: AGCCTGTAGTGAGGCAGCAT  Probe: TGCGTGTCTGAGGAACATCCG | (Jaafar, ChettriDalsgaard, Al-Jubury, Kania, Skov and Buchmann, 2015) |
| *IL-17A/F2a*  *AJ580842* | 158 | Fwd: TCAAAAGCAACGTGTCGAAG  Rev: TCCCTCTGATTCCTCTGTGG  Probe: TATGCTGCTGGGCCTGACCA | (Jaafar *et al.,* 2015) |
| *IL-17c1*  *CAW30792* | 138 | Fwd: CTGGCGGTACAGCATCGATA  Rev: GAGTTATATCCATAATCTTCGTATTCGGC  Probe: CGTGATGTCCGTGCCCTTTGACGATG | (Chettri *et al.,* 2014) |
| *IL-17c2*  *CAW30793* | 134 | Fwd: CTGGCGGTACAGCATCGATA  Rev: CAGAGTTATATGCATGATGTTGGGC  Probe: CGTGGTGTCCAGGCCCTTTAATGATG | (Chettri *et al.,* 2014) |
| *IL-22*  *AM748537* | 64 | Fwd: ATGACCACCACCACAGCATT  Rev: ATTCCTTTCCCCTCCTCCAT  Probe: CTTTCCGCAAGAAGTTGTCCGAG | (Olsen, Kania, Heinecke, Skjoedt, Rasmussen and Buchmann, 2011) |
| *Lysozyme*  *X59491* | 188 | Fwd: GAAACAGCCTGCCCAACT  Rev: GTCCAACACCACACGCTT  Probe: ATACCCAGGCCACCAACCGCAACAC | (Chettri, Raida, Kania and Buchmann, 2012) |
| *SAA*  *AM422446* | 79 | Fwd: GGGAGATGATTCAGGGTTCCA  Rev: TTACGTCCCCAGTGGTTAGC  Probe: TCGAGGACACGAGGACTCAGCA | (Skov, Kania, Holten-Andersen, Fouz and Buchmann, 2012) |
| *TCR-β*  *AF329700* | 73 | Fwd: TCACCAGCAGACTGAGAGTCC  Rev: AAGCTGACAATGCAGGTGAATC  Probe: CCAATGAATGGCACAAACCAGAGAA | (Raida and Buchmann, 2007) |
| *TGF-β1a*  [*X99303*](http://www.ncbi.nlm.nih.gov/entrez/viewer.fcgi?db=nucleotide&val=1478246) | 75 | Fwd: TCTGAATGAGTGGCTGCAAG  Rev: GGTTTCCCACAATCACAAGG  Probe: CTGGAGAGGAGCAGGGATTCCAAT | (Raida and Buchmann, 2007) |
| *TNF-α1 & TNF-α2*  *AJ277604 / AJ401377* | 75 | Fwd: GGGGACAAACTGTGGACTGA  Rev: GAAGTTCTTGCCCTGCTCTG  Probe: GACCAATCGACTGACCGACGTGGA | (Raida and Buchmann, 2008) |
| *IAG52A (I. multifiliis.)*  *AF324424* | 240 | Fwd: TTGGAACTGAAACTAACACAGCC  Rev: CTCCACCTGCAATTGCGGTA  Probe: TGCTGCTGCTTTCGTTCCTGGTGC | This Study |

**References to Table S1**

Chettri, J.K., Kuhn, J.A., Jaafar, R.M., Kania, P.W., Moller, O.S. & Buchmann, K. (2014) Epidermal response of rainbow trout to Ichthyobodo necator: immunohistochemical and gene expression studies indicate a Th1-/Th2-like switch. *Journal of Fish Diseases,* *37***,** 771-783.

Chettri, J.K., Raida, M.K., Kania, P.W. & Buchmann, K. (2012) Differential immune response of rainbow trout (Oncorhynchus mykiss) at early developmental stages (larvae and fry) against the bacterial pathogen Yersinia ruckeri. *Developmental & Comparative Immunology,* *36,* 463-474.

Ingerslev, H.-C., Pettersen, E.F., Jakobsen, R.A., Petersen, C.B. & Wergeland, H.I. (2006) Expression profiling and validation of reference gene candidates in immune relevant tissues and cells from Atlantic salmon (Salmo salar L.). *Molecular Immunology,* *43,* 1194-1201.

Jaafar, R.M., Chettri, J.K., Dalsgaard, I., Al-Jubury, A., Kania, P.W., Skov, J. & Buchmann, K. (2015) Effects of adjuvant Montanide™ ISA 763 A VG in rainbow trout injection vaccinated against Yersinia ruckeri. *Fish & Shellfish Immunology,* *47***,** 797-806.

Marana, M.H., Chettri, J.K., Salten, M.B., Bach-Olesen, N.E., Kania, P.W., Dalsgaard, I. & Buchmann, K. (2020) Primary immunization using low antigen dosages and immunological tolerance in rainbow trout. *Fish & Shellfish Immunology,* *105,* 16-23.

Olsen, M.M., Kania, P.W., Heinecke, R.D., Skjoedt, K., Rasmussen, K.J. & Buchmann, K. (2011) Cellular and humoral factors involved in the response of rainbow trout gills to *Ichthyophthirius multifiliis* infections: Molecular and immunohistochemical studies. *Fish & Shellfish Immunology,* *30,* 859-869.

Purcell, M.K., Kurath, G., Garver, K.A., Herwig, R.P. & Winton, J.R. (2004) Quantitative expression profiling of immune response genes in rainbow trout following infectious haematopoietic necrosis virus (IHNV) infection or DNA vaccination. *Fish & Shellfish Immunology,* *17,* 447-462.

Raida, M.K. & Buchmann, K. (2007) Temperature-dependent expression of immune-relevant genes in rainbow trout following *Yersinia ruckeri* vaccination. *Diseases of Aquatic Organisms,* *77,* 41-52.

Raida, M.K. & Buchmann, K. (2008) Bath vaccination of rainbow trout (*Oncorhynchus mykiss* Walbaum) against *Yersinia ruckeri*: effects of temperature on protection and gene expression. *Vaccine,* *26,* 1050-1062.

Raida, M.K. & Buchmann, K. (2009) Innate immune response in rainbow trout (*Oncorhynchus mykiss*) against primary and secondary infections with *Yersinia ruckeri* O1. *Dev Comp Immunol,* *33,* 35-45.

Skov, J., Chettri, J.K., Jaafar, R.M., Kania, P.W., Dalsgaard, I. & Buchmann, K. (2018) Effects of soluble immunostimulants on mucosal immune responses in rainbow trout immersion-vaccinated against Yersinia ruckeri. *Aquaculture,* *492,* 237-246.

Skov, J., Kania, P.W., Holten-Andersen, L., Fouz, B. & Buchmann, K. (2012) Immunomodulatory effects of dietary beta-1,3-glucan from Euglena gracilis in rainbow trout (Oncorhynchus mykiss) immersion vaccinated against Yersinia ruckeri. *Fish & Shellfish Immunology,* *33,* 111-120.

Xueqin, J., Kania, P.W. & Buchmann, K. (2012) Comparative effects of four feed types on white spot disease susceptibility and skin immune parameters in rainbow trout, *Oncorhynchus mykiss* (Walbaum). *Journal of Fish Diseases,* *35***,** 127-135.
